# The psychosocial well-being of an individual is a driver for illegal immigration: a clinical case study

**DOI:** 10.1192/j.eurpsy.2024.1088

**Published:** 2024-08-27

**Authors:** I. Bouguerra, A. Touiti, A. Hajri, A. Maamri, H. Zalila

**Affiliations:** ^1^Outpatient Psychiatric and emergency Department, Razi Hospital, Manouba; ^2^Faculty of Medicine, Tunis El Manar University, Tunis, Tunisia

## Abstract

**Introduction:**

The European Border and Coast Guard Agency (Frontex) detected 330,000 irregular border crossings last year.Tunisian nationals are among the top three nationalities reported.While there are many reasons for illegal immigration,the social driver is one of the most important to study.

**Objectives:**

To highlight the role of the social environment in promoting illegal immigration and its impact on the psychosocial well-being of individuals in Tunisia through a clinical case study.

**Methods:**

we reported the clinical case of a 32 years old tunisian patient who was diagnosed with severe major depressive disorder and post traumatic stress disorder after an illigale immigration to Europe.

**Results:**

A 32-year-old Tunisian man from Tataouine,a region in southern Tunisia,was the subject of this case study.He was the youngest of four siblings,had a secondary education,and worked as a shepherd.His socio-economic status was moderate,unstable and seasonal.He had already attempted to immigrate to Italy twice illegally,by sea,but had been deported both times.The patient sought consultation for depressive symptoms.In January 2023,he made a third attempt to immigrate to Europe,this time by plane.He traveled with two of his cousins,aged 18 and 20,and paid 22,000 Tunisian dinars for the trip.Both cousins died during the journey,one from hypothermia and the other from police pursuit.The patient was deported again and was diagnosed with post-traumatic stress disorder (PTSD) and a major depressive episode.

**Table:** 

Hamilton Depression scale	PTSD checklist scale	Rosemberg self esteem scale
22 severe depression	66 sup 44	Poor self esteem 21

Tataouine is konwn as “little Paris” a region in the south-east which,according to the National Institute of Statistics,had 71 emigrants/1000 inhabitants or 7.1% of the population.Where the society promotes youth immigration through societal values.

**Conclusions:**

The social environment in Tataouine,which has a high rate of emigration,promotes the idea that immigration is a way to achieve social status and economic security. This can lead to young people feeling pressured to immigrate, even if they are not prepared for the risks involved.

**Disclosure of Interest:**

None Declared

